# Prevalence of musculoskeletal disorders and rheumatic diseases in the indigenous Qom population of Rosario, Argentina

**DOI:** 10.1007/s10067-016-3192-2

**Published:** 2016-02-06

**Authors:** Rosana Quintana, Adriana M. R. Silvestre, Mario Goñi, Vanina García, Nora Mathern, Marisa Jorfen, Julio Miljevic, Daniel Dhair, Matias Laithe, Silvana Conti, Fadua Midauar, Maria Celeste Martin, Maria Cecilia Barrios, Romina Nieto, Cristina Prigione, Alvaro Sanabria, Viviana Gervasoni, Emilio Grabbe, Romina Gontero, Ingris Peláez-Ballestas, Bernardo A. Pons-Estel

**Affiliations:** Hospital Provincial de Rosario, Leandro N. Alem 1450, Rosario, Santa Fe Argentina; Ministerio de Salud, Gobierno de la Provincia de Santa Fe, Santa Fe, Argentina; Centro de Especialidades Médicas Ambulatorias de Rosario, Secretaría de Salud Pública, Municipalidad de Rosario, Santa Fe, Argentina; Instituto de Reumatología, Ortopedia y Fisiatría, Rosario, Santa Fe Argentina; Juan Bautista Alberdi Hospital, Rosario, Santa Fe Argentina; Facultad de Ciencias Médicas, Universidad Nacional de Rosario, Rosario, Santa Fe Argentina; José María Cullen Hospital, Santa Fe, Argentina; Hospital General de México “Dr. Eduardo Liceaga”, Mexico City, Mexico

**Keywords:** COPCORD methodology, Indigenous people, Latin America, Prevalence, Qom, Rheumatic diseases

## Abstract

This study aimed to estimate the prevalence of musculoskeletal disorders and rheumatic diseases among the indigenous Qom (Toba) population in the city of Rosario, Santa Fe, Argentina. An analytical cross-sectional study using methodology of the Community Oriented Program for the Control of Rheumatic Diseases (COPCORD) was performed. Subjects ≥18 years of age were interviewed by advanced students of medicine and nursing, bilingual translator-facilitators, and coordinators. Individuals with musculoskeletal pain (positive cases) were evaluated sequentially for 7 days by internists and rheumatologists for diagnosis and treatment. The study included 1656 individuals (77 % of the census population). Of these, 1020 (61.5 %) were female, with mean age of 35.3 (SD 13.9) years, and 1028 (62.0 %) were bilingual. The public health care system covers 87.1 % of the population. Musculoskeletal pain in the previous 7 days and/or at some time during their life was present in 890 subjects (53.7 %). Of those with pain in the last 7 days, 302 (64.1 %) subjects had an Health Assessment Questionnaire Disability Index (HAQ-DI) score ≥0.8. The most frequent pain sites were lumbar spine (19.3 %), knees (13.0 %), and hands (12.0 %). The prevalence of rheumatic diseases was as follows: mechanical back pain (20.1 %), rheumatic regional pain syndrome (2.9 %), osteoarthritis (4.0 %) rheumatoid arthritis (2.4 %), inflammatory back pain (0.2 %), systemic sclerosis (0.1 %), Sjögren syndrome (0.1 %), fibromyalgia (0.1 %), mixed connective tissue disease (0.06 %), and systemic lupus erythematosus (0.06 %). The prevalence of musculoskeletal disorders was 53.7 % and rheumatic diseases 29.6 %. Rheumatoid arthritis prevalence was 2.4 % using COPCORD methodology, one of the highest reported at present.

## Introduction

Rheumatic diseases represent a broad and heterogeneous group of pathologies related to frequent disabilities and impaired quality of life, some associated with increased mortality. In 2000, the World Health Organization (WHO) raised the priority for musculoskeletal (MSK) disorders as a major health issue owing to secondary disability, and through an increased use of health resources, officially launched what is known as the Bone and Joint Decade (2000–2010) [1].

The WHO and the International League of Associations for Rheumatology (ILAR) developed the Community Oriented Program for the Control of Rheumatic Diseases (COPCORD) for the purpose of obtaining data on MSK disorders and rheumatic diseases in developing countries, in a simple and economical way. The COPCORD methodology has been used in several Asian and Middle Eastern countries, as well as in Australia [2–5]. Additionally, several countries in Latin America, such as México [6], Guatemala [7], Cuba [8], Peru [9], Venezuela [10], and Brazil [11], have also implemented this methodology.

It is estimated that 400 different indigenous groups live throughout Latin America. The countries with the highest proportion of indigenous populations are Bolivia, Guatemala, and Peru, following by Belize, Mexico, and Honduras [12]. Owing to Latin America’s characteristic ethnicity and sociodemographic profile (a mostly mestizo population with low socioeconomic status) and considering that people of indigenous ancestry often show a worse prognosis of some diseases, such as systemic lupus erythematosus (SLE) and rheumatoid arthritis (RA), two multinational and multicenter study groups were created, the Latin American Group for the Study of Lupus (Grupo Latino Americano De Estudio del Lupus; GLADEL) and the Latin American Group for the Study of Rheumatoid Arthritis (Grupo Latino Americano de estudio De Artritis Reumatoide; GLADAR). These groups have reported significant differences especially in terms of age at disease onset, delay in diagnosis, clinical features, and disease activity and severity [13–16].

Because of a need for reliable data regarding MSK disorders and rheumatic diseases in the Latin American indigenous population, the Latin American Group for the Study of Rheumatic Diseases in Original Populations was created (Grupo Latino Americano De estudio de Enfermedades Reumáticas en Pueblos Originarios; GLADERPO). The main purpose in the first phase was to carry out epidemiological, anthropological, and genetic studies, so as to trigger intervention processes in the affected populations during the second phase. Currently, studies in several indigenous populations from Argentina, Mexico, and Venezuela are being conducted.

There are several groups of indigenous people in Argentina. According to the latest census in 2010, it is estimated that nearly 1,000,000 inhabitants identified themselves as belonging to or descendants of an indigenous ethnic group [17]. The Qom or Toba people belong to the ethnic group known as Guaycurú. They originally lived in northern Argentina and expanded to the neighboring countries of Bolivia and Paraguay, called the “Gran Chaco” region. With the arrival of Europeans and subsequent land-grabbing and deforestation, native people were forced to leave their natural environment and relocate to various areas throughout Argentina, among these, the city of Rosario. Owing to its social inclusion policies and relative geographical proximity to Qom native homelands, Rosario has become one of the most densely populated nuclei apart from their places of origin [18].

The aim of this study is to estimate the prevalence of MSK disorders and rheumatic diseases among the Qom population living in Rosario, Santa Fe Province, Argentina.

## Materials and methods

A community-based epidemiological, analytical cross-sectional study was carried out using the methodology of COPCORD in the Qom population living in Rosario, Argentina.

### Study population

We included individuals ≥18 years of age who self-identified as Qom and had been resident for at least 6 months in Rosario. Rosario is a city located 306 km north of the capital of Argentina, with a population of more than 1,190,000 [17]. The Qom population is located in the following three districts: Northern (borough of Travesía), Northwest (borough of Pumitas), and Western (borough of Rouillón). There were no official data available on the number of individuals residing in the city, owing to high migration. Therefore, it was necessary to conduct a population census and map the collected data.

### Survey

The COPCORD questionnaire, which has been previously transculturally adapted and validated, served as the data collection tool [19]. The questionnaire was administered by advanced students of medicine and nursing who had been trained. These students were accompanied by bilingual Spanish/Qom translators-facilitators and coordinators. The COPCORD questionnaire consists of the following four sections: an explanatory section; a section that includes sociodemographic data, work history, and self-reported illnesses; and a section to identify MSK pain in the last 7 days or at some time during the subject’s lifetime, which queries features such as pain intensity, physical limitation, adaptation, and help-seeking behavior and medical and/or traditional treatment. Finally, the questionnaire includes a section designed to assess functional capacity, measured by the short and validated Health Assessment Questionnaire Disability Index (HAQ-DI) [20].

In addition, a survey using socioeconomic and health indicators was specifically designed by primary health care teams. The survey included time of residence in the neighborhood, degree of formal education, health coverage, monthly income, and living conditions.

### Study design

At baseline, the strategy included a pilot study with 103 Qom individuals from the borough of Pumitas; these subjects were excluded from the extended epidemiological study [19].

Implementation of the survey was done through door-to-door home visits in the three districts. If the individual was not at home, visits were repeated at different times and days of the week, for at least five times; after this, the participant was considered “absent.”

Home visits began with a presentation given by the Qom facilitator, who explained the purpose of the study, participant confidentiality, and the future use of the data. People were invited to participate and an informed consent form was provided, with enough time given for individuals to read and understand it. If the individual was illiterate, the consent procedure was verbally explained. Signing of informed consent was done in front of witnesses, a translator/facilitator, and a representative of the Provincial Ministry of Health.

All subjects reporting pain, swelling, or stiffness over the last 7 days or at any point during their lifetime were considered positive for clinical examination by an internal medicine specialist. If any examined subject had a clinical evaluation suggesting a rheumatic disease, they were referred to a rheumatologist within the next 7 days for diagnosis and treatment. The study lasted a total of 18 months.

A rheumatologist provided the diagnosis according to standardized criteria for RA [21], SLE [22], systemic sclerosis (SSc) [23], Sjögren syndrome (SS) [24], mixed connective tissue disease (MCTD) [25], inflammatory back pain [26], osteoarthritis (OA) [27], back pain [28], fibromyalgia [29], and rheumatic regional pain syndrome (RRPS) [30]. In the case of back pain or probable RRPS, validated questionnaires were administered [6, 30, 31]. These disorders, which could not be included in the previously named categories, were designated unknown MSK disorders (UMSK), according to the International Classification of Diseases, 10th Revision (ICD-10), published by the WHO [32].

### Ethical aspects

The study was approved on December 22, 2010 by the Research Ethics Committee of the Health Department of the Municipality of Rosario (Resolution No. 1659/2009), with the support of the Ministry of Health (resolutions 1619/2010 and 0127/2011) and the Ministry of Social Development of the Province of Santa Fe, the Association of Rheumatology of Santa Fe, Faculty of Medical Sciences, National University of Rosario, and representatives of Qom city organizations. The study was registered (registration order number 13) with the Provincial Bioethics Committee of the Province of Santa Fe on July 5, 2012.

### Statistical analysis

The data were entered via a web interface into a database designed for the study. All analyses were conducted using Stata v.11.0 (StataCorp, College Station, TX, USA). A descriptive analysis with measures of central tendency and dispersion for continuous variables was performed. Absolute and relative frequencies for categorical variables were estimated. Bivariate analysis was performed for each study variable, using one-way and two-way analysis of variance (ANOVA) for continuous variables and chi-squared for nominal or categorical ordinal variables. A comparative analysis of variable frequencies, with a significance level of 0.05, was performed.

A logistic regression in four models, both block and forward stepwise, was conducted, using as dependent variables: (1) rheumatic disease plus UMSK; (2) rheumatic disease, excluding UMSK; (3) rheumatic inflammatory disease (RA, undifferentiated arthritis, SLE, MCTD, inflammatory back pain, SS, and SSc); and (4) noninflammatory rheumatic disease (OA, mechanical back pain, fibromyalgia, and RRPS). The WHO ICD-10 was used to create these categories [32].

Independent variables included those with clinical relevance and/or statistical significance (*p* ≤ 0.05), sociodemographic variables, currently working, work involving loads, repetitive work, pain in the last 7 days, pain intensity, some kind of treatment received, and functional disability measured by HAQ-DI. This analysis aimed to identify those variables associated with the presence of rheumatic disease, which could be used as proxy predictors for derivation, from primary to specialist care. The HAQ-DI score was categorized according to values, with a cutoff of 0.8 [20].

## Results

The Qom population in our study included a total of 2157 individuals living stably in the three districts of Rosario and were all ≥18 years. Of these, 1759 (81.5 %) participated in the epidemiological study, 103 in the pilot study, and 1656 in the extended study. Three-hundred and twenty-two (14.9 %) individuals were considered absent and 76 (3.5 %) did not agree to participate.

Of the total number of individuals included in the study (*n* = 1656), 1020 (61.5 %) were female, with mean age of 35.3 years (SD 13.9). Birthplace was reported as Chaco Province by 1356 (81.8 %) individuals. A total of 1636 (98.7 %) participants spoke Spanish and 1028 (62.0 %) spoke the Qom language.

The majority of surveyed individuals declared that they had Qom ancestors, the mother being Qom in 93.1 % and the father being Qom in 84.2 %. A total of 1381 (83.4 %) individuals reported some level of education, but only 338 (20.1 %) had completed primary school, 54 (3.2 %) secondary school, and 3 (0.1 %) the tertiary level. Only two individuals (0.1 %) reported incomplete university studies. At the time of the survey, 1060 (64.0 %) individuals were occupationally active, but only 106 (10.1 %) had formal employment. With respect to the type of work, 646 individuals (39.0 %) reported work involving loads (≥4 kg) and 639 (38.5 %) reported their work involved repetitive tasks. In relation to health coverage, 1444 (87.1 %) participants were users of the public health system (Table 1).

**Table 1:** Sociodemographic characteristics of the Qom population

Sociodemographic variables	*N* = 1656 *n* (%)
Female	1020 (61.5)
Age, mean (SD; range), years	35.3 (13.9; 18–105)
Marital status^a, (59)^	
With partner	1064 (64.2)
Without partner	481 (29.0)
Separated/divorced	52 (3.1)
Place of birth (province)	
Chaco	1356 (81.8)
Santa Fe	266 (16.0)
Formosa	17 (1.0)
Buenos Aires	11 (0.6)
Santiago del Estero	3 (0.1)
Misiones	2 (0.1)
Language	
Spanish	1636 (98.7)
Qom	1028 (62.0)
Ancestor Qom	
Mother	1542 (93.1)
Father	1396 (84.2)
Level of education	
Primary school (completed)	338 (20.1)
Secondary school (completed)	54 (3.2)
Tertiary school (completed)	3 (0.1)
University studies (incomplete)	2 (0.1)
Current work	1060 (64.0)
Load work (≥4 kg)	646 (39.0)
Repetitive work	639 (38.5)
Type of social health coverage^a, (5)^	
Public	1444 (87.1)
Private	181 (10.8)
PAMI	26 (1.5)

*SD* standard deviation, *PAMI* state social health coverage

^a^Missing data

Self-reported illnesses, in order of frequency, were gastritis 378 (21.5 %), arterial hypertension 318 (18.1 %), Chagas disease 273 (15.5 %), venous insufficiency 218 (12.4 %), heart disease 125 (7.1 %), obesity 119 (6.8 %), diabetes mellitus 112 (6.4 %), and dyslipidemia 61 (3.5 %). A total of 104 individuals (6.3 %) reported having a previous diagnosis of rheumatic disease, the most common being OA (20, 19.2 %) and RA (19, 18.2 %).

**Fig. 1:**
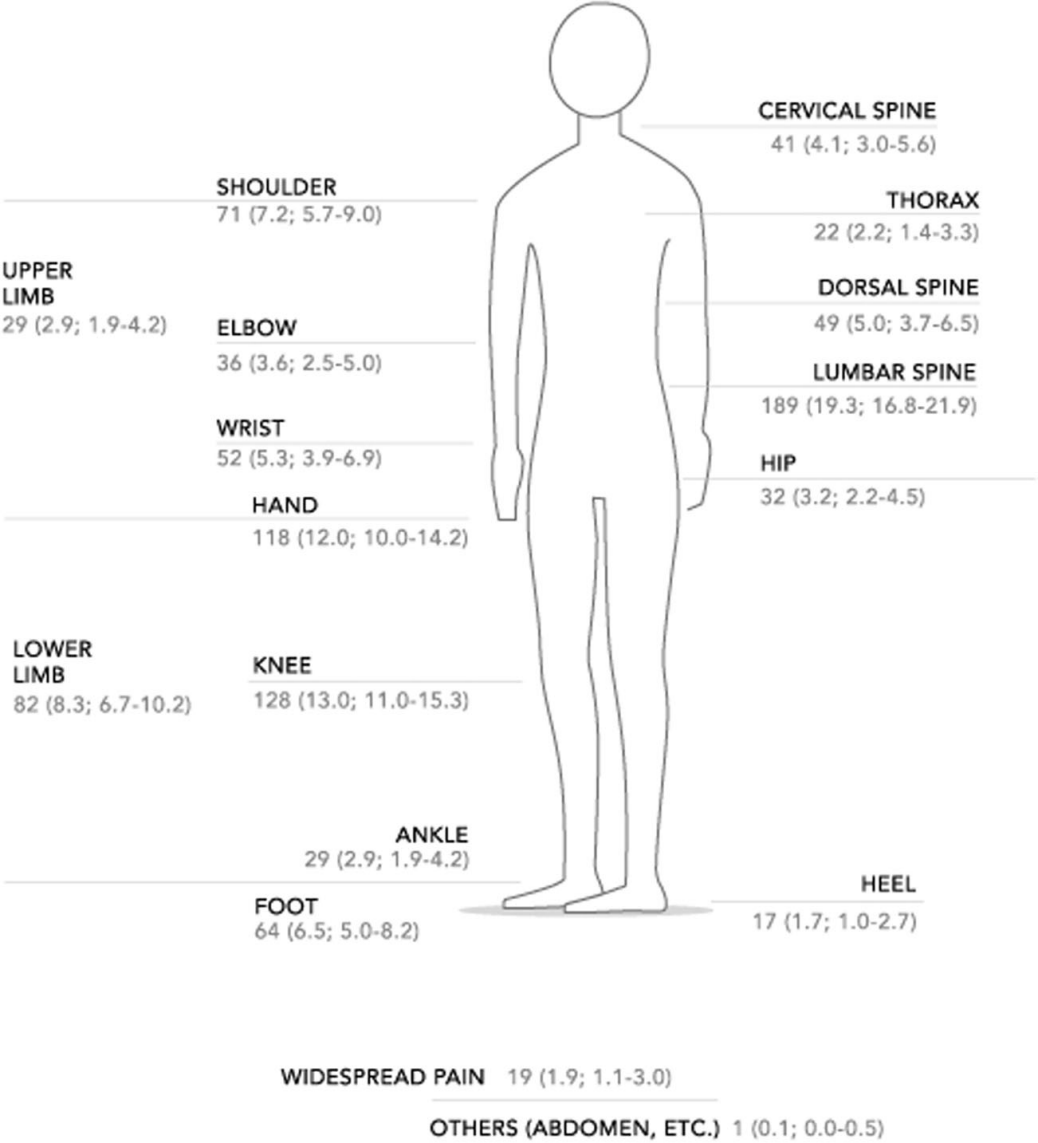
Sites of musculoskeletal pain in the last 7 days

MSK pain was reported by 890 (53.7 %) participants. Of 471 (52.9 %) individuals who had been referred in the last 7 days, 376 (79.8 %) had no associated trauma. Pain at some point in life (historical pain) was reported by 514 (57.7 %) individuals. Both pain in the last 7 days and historical pain were present in 278 (31.2 %) individuals. Within the subgroup of individuals with pain during the last 7 days, 364 (77.3 %) had severe pain (reported as “quite a lot” and “a lot”). Regarding pain adaptation (coping), 14 (3.0 %) reported not being able to cope, 226 (48.0 %) partially able to cope, and 231 (49.0 %) adequate coping. A total of 81 (17.2 %) had actual physical limitations in performing their daily tasks, and 302 (64.1 %) individuals had an HAQ-DI ≥ 0.8 (Table 2). The most common sites of MSK pain recorded in the last 7 days are shown in Fig. 1, with the most representative being lumbar spine in 189 (19.3 %) participants, knees in 128 (13.0 %), and hands in 118 (12.0 %).

**Table 2:** Description of musculoskeletal pain in the last 7 days

Musculoskeletal pain	*N* = 471 *n* (%)
Pain intensity	
None	27 (5.8)
Little	2 (0.4)
Regular	78 (16.5)
Quite a lot	162 (34.4)
A lot	202 (42.9)
Adaptation to pain	
Not being able to cope	14 (3.0)
Partially able to cope	226 (48.0)
Adequate coping	231 (49.0)
Physical limitation	
Past physical limitation	71 (15.1)
Current physical limitation	81 (17.2)
Never had physical limitation	319 (67.7)
HAQ-DI score ≥ 0.8, *n* (%; 95 % IC)	302 (64.1; 59.6–68.4)

*HAQ-DI* Health Assessment Questionnaire Disability Index

Of all individuals with MSK pain (*n* = 890), 289 (32.4 %) attended a primary care center to seek assistance, 6 (0.6 %) turned to traditional medicine, 75 (8.4 %) received another type of support, and 378 (42.4 %) reported not having sought any assistance. A total of 142 (15.9 %) individuals did not answer this question. A total 381 of 890 (42.8 %) positive cases had received some kind of treatment; 281 (73.7 %) received nonsteroidal anti-inflammatory drugs (NSAIDs), 16 (4.1 %) corticosteroids, and 10 (2.6 %) disease-modifying anti-rheumatic drugs (DMARDs).

After evaluation by a rheumatologist, 752 (45.4 %) MSK disorders and rheumatic diseases were diagnosed. The prevalence of rheumatic diseases, excluding UMSK, was 29.7 %. The prevalence of inflammatory rheumatic diseases was 5.5 % and that of noninflammatory rheumatic diseases was 26.3 %. The prevalence of each disease was as follows: mechanical back pain (20.1 %), RRPS (2.9), OA (4.0 %), RA (2.4 %), undifferentiated arthritis (0.3 %), inflammatory back pain (0.2 %), SSc (0.1 %), primary SS (0.1 %), fibromyalgia (0.1 %), MCTD (0.06 %), and SLE (0.06 %). A total of 260 (15.7 %) cases were classified as UMSK (Table 3).

**Table 3:** Prevalence of musculoskeletal disorders and rheumatic diseases in 1656 Qom’s population

Musculoskeletal disorders and rheumatic disease	Total *n* (%; 95 % CI) *n* = 1656	Female *n* (%; 95 % CI) *n* = 1020	Male *n* (%; 95 % CI) *n* = 636	*P* value^a^
Osteoarthritis	67 (4.0; 3.1–5.1)	41 (4.0; 2.8–5.4)	26 (4.0; 2.6–5.9)	0.5
Rheumatic regional pain syndromes	48 (2.9; 2.1–3.8)	44 (4.3; 3.1–5.7)	4 (0.6;0.1–1.6)	<0.01
Undifferentiated arthritis	5 (0.3; 0.09–0.7)	4 (0.3; 0.1–1.0)	1 (0.1; 0.003–0.8)	0.3
Mechanical back pain	333 (20.1; 18.2–22.1)	186 (18.2; 15.9–20.7)	147 (8.8; 7.5–10.3)	0.03
Inflammatory back pain	4 (0.2; 0.03–0.5)	0	4 (0.6; 0.1–1.6)	–
Rheumatoid arthritis	40 (2.4; 1.7–3.2)	35 (3.4; 2.4–4.7)	5 (0.7; 0.2–1.8)	<0.01
Systemic sclerosis (scleroderma)	2 (0.1; 0.01–0.4)	1 (0.09; 0.002–0.5)	1 (0.1; 0.003–0.8)	0.6
Primary Sjögren syndrome	2 (0.1; 0.01–0.4)	1 (0.09; 0.002–0.5)	1 (0.1; 0.003–0.8)	0.6
Fibromyalgia	2 (0.1; 0.01–0.4)	1 (0.09; 0.002–0.5)	1 (0.1; 0.003–0.8)	0.6
Mixed connective tissue disease	1 (0.06; 0.001–0.3)	1 (0.09; 0.002–0.5)	0	–
Systemic lupus erythematosus	1 (0.06; 0.001–0.3)	1 (0.09; 0.002–0.5)	0	–

^a^Chi-squared test (dichotomous)

A comparison between patients with diagnosed rheumatic disease and UMSK versus healthy subjects (negative survey) was performed. Variables with observed statistical significance were age (37.3 vs 33.7 years, *p* < 0.01), current work (68.0 vs 60.6 %, *p* < 0.01), load work (43.8 vs 34.9 %, *p* < 0.01), repetitive work (43.2 vs 34.7 %, *p* < 0.01), presence of pain in the last 7 days (62.3 vs 0.2 %, *p* < 0.01), severe pain (62.6 vs 0.3 %, *p* < 0.01), treatment received (50.3 vs 0.2 %, *p* < 0.01), and HAQ-DI ≥ 0.8 (56.2 vs 8.6 %, *p* < 0.01) (Table 4).

**Table 4:** Comparing individuals with rheumatic disease and unknown musculosketal disorders and healthy subjects (survey negative)

Variables	Rheumatic disease and UMKS *n* = 752 *n* (%)	Healthy subjects (survey negative) *n* = 904 *n* (%)	*P* value
Female	443 (58.9)	577 (63.8)	0.04
Age, median (SD)	37.3 (14.1)	33.7 (13.5)	<0.01
Current work	512 (68.0)	548 (60.6)	<0.01
Load work ≥ 4 kg	330 (43.8)	316 (34.9)	<0.01
Repetitive work	325 (43.2)	314 (34.7)	<0.01
Pain in the last 7 days	469 (62.3)	2 (0.2)	<0.01
Severe pain	471 (62.6)	3 (0.3)	<0.01
Treatment received	379 (50.3)	2 (0.2)	<0.01
Physical limitation	115 (15.2)	1 (0.1)	0.08
HAQ-DI ≥ 0.8	423 (56.2)	78 (8.6)	<0.01

*UMSK* unknown MSK disorders, *SD* standard deviation, *HAQ-DI* Health Assessment Questionnaire Disability Index

In comparing patients with diagnosed rheumatic diseases (excluding UMSK) versus healthy subjects (negative survey), variables with statistical significance were current work (67.9 vs 60 %, *p* < 0.01), load work (44.2 vs 34.9 %, *p* < 0.01), repetitive work (43.8 vs 34.7, *p* < 0.01), pain in the last 7 days (64.6 vs 0.2 %, *p* < 0.01), treatment received (54.8 vs 0.2 %, *p* < 0.01), severe pain (64.2 vs 0.3, *p* < 0.01), and HAQ-DI ≥ 0.8 (64.2 vs 8.6 %, *p* < 0.01).

Comparing patients with inflammatory rheumatic diseases versus healthy subjects (negative survey), variables with statistical significance were pain in the last 7 days (70.9 vs 0.2 %, *p* < 0.01), severe pain (70.9 vs 0.2 %, *p* < 0.01), treatment received (81.8 vs 0.2 %, *p* < 0.01), and HAQ-DI ≥ 0.8 (81.8 vs 8.6 %, *p* < 0.01).

In the comparison between patients with noninflammatory rheumatic diseases versus healthy subjects (negative survey), variables with statistical significance were female sex (63.8 vs 57.2 %, *p* < 0.01), current work (68.4 vs 60.6 %, *p* < 0.01), load work (45.7 vs 34.9 %, *p* < 0.01), repetitive work (45.3 vs 34.7 %, *p* < 0.01), pain in the last 7 days (63.9 vs 0.2 %, *p* < 0.01), severe pain (64.3 vs 0.3 %, *p* < 0.01), treatment received (51.6 vs 0.2 %, *p* < 0.01), and HAQ-DI ≥ 0.8 (49.2 vs 8.6 %, *p* < 0.01).

Four models for multiple logistic regression analysis were performed. The analysis aimed at identifying those variables that could be used by the primary care physician as proxy for referring patients to the specialist. The presence of pain in the last 7 days and HAQ-DI ≥ 0.8 were the variables significantly more relevant in the four models, model 1, rheumatic disease plus UMSK: pain OR 549.2, 95 % confidence interval (CI) 134.0–2250.1, *p* < 0.01 and HAQ-DI OR 7.26, 95 % CI 4.8–10.9, *p* < 0.01; model 2, rheumatic disease excluding UMSK: pain OR 516.1, 95 % CI 123.6–2154.2, *p* < 0.01 and HAQ-DI OR 8.5, 95 % CI 5.3–13.8, *p* < 0.01; model 3, inflammatory rheumatic disease: pain OR 206.4, 95 % CI 21.9–1944.9, *p* < 0.01 and HAQ-DI OR 8.28, 95 % CI 1.1–61.0, *p* < 0.03; and finally, model 4, noninflammatory rheumatic disease: pain OR 513.7, 95 % CI 122.9–2146.4, *p* < 0.01 and HAQ-DI OR 8.5, 95 % CI 5.2–13.8, *p* < 0.01.

## Discussion

The most relevant findings of this study were the high prevalence of RA, and a female to male ratio of 7:1, similar to the one published by GLADAR [15]. On the basis of the overall cases reported, 21 (52.5 %) were diagnosed during the present study and 19 (47.5 %) had a prior diagnosis. In addition, undifferentiated arthritis was diagnosed in six (0.3 %) individuals; these participants were not taken into account in the clinical follow-up data used in this paper.

The prevalence of RA, found here using COPCORD methodology, was surpassed by that reported in 2011 for the Yucatán Península of Mexico (2.8 %) [6]. Few studies have been published on the RA prevalence among indigenous people using this type of methodology. In a recent study in Guatemala [7], two regions were compared, one urban and the other rural (where more than 60 % of inhabitants belong to a native community, the Kaqchikel). The prevalence of RA in the latter region was 0.8 versus 0.5 % in the urban area [7].

Another study carried out using COPCORD methodology in an indigenous community in Australia (Yarrabah population) did not show any evidence of RA cases [33]. These differences may be related to genetic and/or environmental issues. It has been stated that indigenous Australians do not present any evidence of RA [34], different to some native communities of North America where the prevalence of RA reaches 5–7 %; the most representative include the Chippewa (6.8–7.1 %), Pima (5.3 %), Yakima (3.4 % in females), and Tlingit (2.4 %) communities [35]. Furthermore, in these communities, the presentation of RA is more aggressive and with higher seropositivity.

A study in a native community of Canada (Manitoba’s population) also reported a high prevalence of RA compared with the overall white population in the same area. These data were collected from a database of visit records at a reference site (on-request visits), where it was observed that nearly twice the number of records of medical attention belonged to patients who were part of the native community (40.9 vs 25.9 %, *p* = 0.000) [36].

In Argentina, two studies described the prevalence of RA in the overall population. One study was conducted in Tucumán (northwest region). The data used were obtained from inpatient and outpatient records from public and private health care facilities. A global RA prevalence rate of 1.97 per 1000 inhabitants (0.2 %) was reported. According to its authors, 84 % of the inhabitants were European descendants, whereas indigenous people represented only 10 % [37]. The other study was conducted in Luján, Buenos Aires Province. These data were collected from rheumatologists by a phone survey. The prevalence of RA was 0.9 % [38]; however, participants’ ethnicities were not analyzed.

In our study, the finding involving family members with RA was relevant. Six families were included; five had two family members (mother and daughter) with a diagnosis of RA, and the other family had three affected members (mother and daughter with RA diagnosis and another daughter with SLE diagnosis). This familial aggregation has not been found in any previous COPCORD study. It has been observed that the chance of developing RA, even of having positive serological results without developing disease, is very high among first-degree family members in North American indigenous populations [39, 40].

Another remarkable finding in the present study was the high prevalence of MSK pain in general (positive cases) of around 53.7 %, and particularly of pain during the last week (52.9 %), related to significant functional disability measured by HAQ-DI. In COPCORD surveys in native communities of Guatemala [7] and Australia [32], the prevalence of MSK pain was of a lesser degree, around 4.5 and 33 %, respectively. Another study carried out in a rural area of Iran showed a pain prevalence of 41.9 % [41]. In relation to studies performed in Latin America, Mexico showed an overall prevalence of 25.5 % [6], Cuba 43.9 % [8], Brazil 30.9 % [11], Venezuela 22.4 % [10], and Peru 46.6 % [9].

Mechanical back pain was the most prevalent identified symptom in this study (20.1 %), mainly in women, comparable to a COPCORD survey performed in rural Iran, with a percentage of 23.4 % [41]. This similarity is probably owing to the type of work performed by study participants, where load work and repetitive tasks were common in both populations. In other COPCORD studies among indigenous people in Guatemala [7] and Australia [33], the percentage of back pain was of a lesser degree, 5 and 14 %, respectively. In additional COPCORD studies in Latin America, Cuba showed 11.6 % [8], whereas this manifestation was not reported in either Peru [9] or Brazil [11]. A study done in Mexico [41] using COPCORD methodology showed back pain prevalence of 8 % and inflammatory back pain of 3 %. In the present study, only 0.2 % of study participants demonstrated characteristics of inflammatory back pain, all of them men.

In our study, the prevalence of RRPS was 2.9 % and significantly more prevalent in women. RRPS was related to the upper limbs, particularly rotator cuff tendinopathy, similar to those published in other COPCORD studies [6–10, 42]. Mexican authors studied the prevalence of RRPS, using COPCORD methodology, in different areas of the country. The global prevalence reported was 5 % but with differences related mainly to work activity [31].

In our study, the prevalence of detected OA was 4 %, comparable with COPCORD studies performed in Brazil (4.1 %) [11] and Guatemala (3.9 %) [7] but of lesser degree than the results of studies in Mexico (10.2 %) [10] and Cuba (20.4 %) [8]. The low prevalence of detected OA among the Qom community may be related to the low mean age of the study population (35.3 years, SD 13.9).

In relation to autoimmune diseases such as SLE and SSc, the prevalence found in our study (0.06 and 0.1 %, respectively) was similar to that found in other COPCORD studies [6, 8–11]. We also reported primary SS (0.1 %) and MCTD (0.06 %) cases, which have not been described previously.

The low prevalence of fibromyalgia in our study (0.06 %) is notable. This disease was not reported in studies in Guatemala [7] or even in Australia [33]. The prevalence of fibromyalgia in Mexico was 1.4 [6] and 2.5 % in Brazil [11]. We believe these differences may be owing to the perception of indigenous people with respect to pain; in other words, they may have greater pain tolerance.

In the present study, no cases of psoriasis, psoriatic arthritis, or gout were found. Toloza et al. [43] reported that the first two pathologies were infrequent among indigenous communities, especially in Central and South America. Comparing differences between countries, the highest prevalence of psoriasis in Argentina, Chile, and Uruguay was observed where the white population was higher. On the other hand, the prevalence of HLA-B27 is lower in Central and South America (<1 %) compared with North America (9–50 %) [39, 43]. More recently, Toloza et al. [44] published a study of an indigenous community in Puno, Bolivia, describing 16 cases of psoriasis and psoriatic arthritis; six of these cases were descended from native people (five from the Quechua community and one from the Aymara community). Those study subjects belonging to indigenous communities denied any cases among family members. Contrary to what was been published previously, this was the first report on the presence of psoriatic arthritis in native communities of South America. In the Australian Yarrabah indigenous community, only four cases of psoriatic arthritis have been detected [33]. Regarding the absence of gout, similar to what was reported in Guatemala [7] of only one identified case, this could be related to the eating habits of native communities in Latin America. On the contrary, among the Australian indigenous community [33], the prevalence of this pathology was 9.7 % in males and 2.9 % in females [45]. According to the authors, this finding was related to alcohol intake, the prevalence of overweight, and metabolic syndrome.

As shown in the multivariable logistic regression analysis, pain during the last 7 days and functional disability as measured by the HAQ-DI should prompt the primary care physician to refer these patients to a rheumatologist for a prompt diagnosis and treatment.

The limitations of our study were as follows: a higher number of females were included, despite designing an intentional search scheme for absent males. Participation by young subjects (mean age 35.3) might be because they are the most stable residents within the Qom community living in Rosario. A known pattern of frequent migration exists between Rosario and the Qom home province of Chaco.

In conclusion, using COPCORD methodology, we found a high prevalence of MSK disorders and rheumatic diseases in an indigenous Qom community, especially mechanical back pain and RA, the latter with a higher reported prevalence. Future studies are needed to characterize RA among the Qom people.

The use of COPCORD methodology to detect MSK disorders and rheumatic diseases in an easy and rapid fashion must be highlighted. It is important to note that clinical data provided by the patient on pain characteristics and functional disability should be the most important aspect for general physicians to take into account when MSK disorders and rheumatic diseases are suspected, for quick referral to a specialist.
